# Evaluating community-wide temporal sampling in passive acoustic monitoring: A comprehensive study of avian vocal patterns in subtropical montane forests

**DOI:** 10.12688/f1000research.141951.2

**Published:** 2024-01-23

**Authors:** Shih-Hung Wu, Jerome Chie-Jen Ko, Ruey-Shing Lin, Chia-Hao Chang-Yang, Hsueh-Wen Chang

**Affiliations:** 1 Department of Biological Sciences, National Sun Yat-sen University, Kaohsiung, 804, Taiwan; 2 Taiwan Biodiversity Research Institute, Nantou, 552, Taiwan; 3 Institute of Ecology and Evolutionary Biology, National Taiwan University, Taipei, 106, Taiwan

**Keywords:** passive acoustic monitoring, vocal activity rate, temporal sampling, Aves

## Abstract

**Background:**

From passive acoustic monitoring (PAM) recordings, the vocal activity rate (VAR), vocalizations per unit of time, can be calculated and is essential for assessing bird population abundance. However, VAR is subject to influences from a range of factors, including species and environmental conditions. Identifying the optimal sampling design to obtain representative acoustic data for VAR estimation is crucial for research objectives. PAM commonly uses temporal sampling strategies to decrease the volume of recordings and the resources needed for audio data management. Yet, the comprehensive impact of this sampling approach on VAR estimation remains insufficiently explored.

**Methods:**

In this study, we used vocalizations extracted from recordings of 12 bird species, taken at 14 PAM stations situated in subtropical montane forests over a four-month period, to assess the impact of temporal sampling on VAR across three distinct scales: short-term periodic, diel, and hourly. For short-term periodic sampling analysis, we employed hierarchical clustering analysis (HCA) and the coefficient of variation (CV). Generalized additive models (GAMs) were utilized for diel sampling analysis, and we determined the average difference in VAR values per minute for the hourly sampling analysis.

**Results:**

We identified significant day and species-specific VAR fluctuations. The survey season was divided into five segments; the earliest two showed high variability and are best avoided for surveys. Data from days with heavy rain and strong winds showed reduced VAR values and should be excluded from analysis. Continuous recordings spanning at least seven days, extending to 14 days is optimal for minimizing sampling variance. Morning chorus recordings effectively capture the majority of bird vocalizations, and hourly sampling with frequent, shorter intervals aligns closely with continuous recording outcomes.

**Conclusions:**

While our findings are context-specific, they highlight the significance of strategic sampling in avian monitoring, optimizing resource utilization and enhancing the breadth of monitoring efforts.

## 1. Introduction

Biodiversity is paramount for the sustainable progression of human society and environmental preservation. It contributes either directly or indirectly to all 17 of the Sustainable Development Goals (SDGs) ( Blicharska *et al*., 2019). Monitoring biodiversity is a crucial endeavor that facilitates the comprehension of the current state, alterations, and trends of biodiversity and evaluates the efficacy of interventions aimed at mitigating biodiversity loss ( Pereira & Davidcooper, 2006). Birds serve as an ideal indicator taxon for monitoring terrestrial biodiversity due to their detectability, identifiability, diversity, widespread distribution, and migratory characteristics ( Fraixedas *et al*., 2020). Bird monitoring can shed light on the effects of habitat loss, deforestation, climate change, invasive species, light pollution, and illegal hunting ( Xu *et al*., 2019; Northrup *et al*., 2019; Dueñas *et al*., 2021; La Sorte *et al*., 2022; Crespo *et al*., 2020; Negret *et al*., 2021), while also highlight the beneficial outcomes of conservation efforts ( Cazalis *et al*., 2020).

Beyond human observations, various technologies including radar, thermal imaging, and passive acoustics have been employed for manual or automatic bird monitoring ( Lahoz-Monfort & Magrath, 2021). In recent years, PAM has seen an upsurge in its use for bird monitoring and research ( Hoefer *et al*., 2023). Thanks to decreasing costs, autonomous recording units (ARUs) can now be extensively deployed across diverse environments, recording considerable volumes of soundscape data. These data provide rich biological information and allow continuous, automated monitoring, thereby significantly increasing both the temporal and spatial coverage of these efforts ( Ross *et al*., 2023; Pérez-Granados *et al*., 2021; Sugai *et al*., 2019; Shonfield & Bayne, 2017). However, the labor-intensive and time-consuming process of identifying species sounds within soundscape data presents a significant bottleneck for PAM utilization. The rise of automated identification tools, such as BirdNET and SILIC, are steadily alleviating this issue ( Kahl *et al*., 2021; Wu *et al*., 2022). Despite these advancements, handling large volumes of audio files remains a considerable challenge due to the high energy requirements for extended ARU field operations, the need for extensive storage space, and lengthy analysis time ( Zwerts *et al*., 2021).

Sampling has been employed as an effective and commonly used method to decrease the amount of recording data. Sampling design can be categorized into four temporal scales: intra-annual, short-term periodic, diel, and hourly. An intra-annual sampling design implies recording during one or several time periods within a year. Many bird species exhibit more frequent vocalization during the breeding season; hence, the majority of studies prefer to conduct PAM surveys within this timeframe of a year ( Campos-Cerqueira & Aide, 2016; Bateman *et al*., 2021; Duchac *et al*., 2020). Alternatively, certain studies opt for acoustic surveys during the non-breeding season, a period characterized by relatively stable detectability and community composition ( Metcalf *et al*., 2021).

Short-term periodic sampling design is often employed in light of limited ARU availability, necessitating a rotational system among diverse survey locations. Typically, after being operated for a predetermined number of days at each location, the ARU is relocated to a subsequent site. This rotation ensures a comprehensive collection of crucial soundscape data from each location throughout the survey season ( Jahn *et al*., 2022; Machado *et al*., 2017). However, avian vocal activity can vary significantly over time ( Pérez-Granados & Schuchmann, 2020), and the rotation of devices leads to asynchronous data collection, potentially increasing variability. Therefore, concentrating the rotation of devices during periods when avian vocal activity shows relatively minor temporal variations can help mitigate this variability, enhancing the comparability of data across different sites. Furthermore, the duration of each deployment during the rotation process is a critical factor affecting sound data collection. Longer deployment durations can dilute data collected under extreme conditions, such as typhoons, but also increase the volume and processing costs of the data. Choosing an appropriate deployment duration to reduce the impact of extreme events while minimizing deployment time presents a significant challenge.

The diel sampling design is typically framed based on the behavioral patterns of the target species. For instance, as Passeriformes frequently vocalize during the dawn and dusk choruses, the recordings are concentrated around these periods ( Alvarez-Berríos *et al*., 2016; Deichmann *et al*., 2017; Rumelt *et al*., 2021). For nocturnal birds, recordings are conducted during the night ( Jahn *et al*., 2022; Wood *et al*., 2020). When the research objective targets one or a few bird species with similar vocal activity patterns, prior research on the vocal behavior of these species is crucial. It aids in planning the recording schedule to coincide with the peak vocalization periods of the target species. However, when the goal encompasses a wide variety of species with different habits, the challenge lies in optimizing recording times to capture the vocal peaks of most species within limited resources.

Hourly sampling design can be categorized into coverage (the proportion of recorded time within an hour) and dispersion (the number of recording segments within an hour). Examples of such strategies might include recording a one-minute segment ( Diepstraten & Willie, 2021) or a fifteen-minute segment ( Pérez-Granados *et al*., 2021) within an hour, or perhaps recording one minute every ten minutes ( Ducrettet *et al*., 2020; Melo *et al*., 2021) or every fifteen minutes ( Yoo *et al*., 2020), and even recording fifteen minutes every half hour ( Favaro *et al*., 2021). There is a trade-off challenge in balancing reduced coverage with adequate acoustic data collection. Regarding dispersion, the critical question is which recording schedule, whether dispersed or concentrated, more accurately reflects the actual scenario. Furthermore, it's important to understand how different bird species respond to varying levels of dispersion. Both coverage and dispersion are significant factors influencing the design of hourly sampling designs.

The vocal activity rate (VAR) of birds, defined as the quantity of vocalizations per unit of time, can be derived from PAM data. VAR is a pivotal metric in acoustic surveys, enabling the estimation of bird abundance or density, which is crucial for monitoring avian population ( Pérez-Granados *et al*., 2019a; Pérez-Granados & Traba, 2021). However, avian vocal activity is modulated by an intricate blend of both exogenous and endogenous factors. This results in diverse vocal patterns that vary by species, sex, age, temporal factors, environmental conditions, site-specific characteristics, habitat types, and social contexts ( Catchpole & Slater 2003; Marques *et al*. 2013; Bruni *et al*., 2014; Digby *et al*., 2014; Symes *et al*., 2022). The VAR is substantially influenced by its temporal sampling design. For studies targeting individual species or a limited group, the temporal sampling can be customized to their specific behaviors ( Pérez-Granados *et al*., 2019b). Yet, when the scope encompasses an entire avian community, an optimally structured survey should capture the most prevalent species and a significant proportion of the less common ones ( Franklin *et al*., 2021). Past research has underscored the profound impact of the recording schedule on assessments of avian community richness and composition. For example, prolonging recording durations generally augments species detection, especially for less common species ( Wood *et al*., 2021; Symes *et al*., 2022). Concentrating recordings during specific time, like dawn, often captures more species but may miss those from distinct functional groups ( Shaw *et al*., 2022). Nevertheless, the effects of temporal sampling designs on the VAR of individual species within a community remain under-investigated.

This study aims to evaluate the impact of various temporal sampling methodologies on the identification of VAR patterns in a biotic community, with a focus on avian communities in subtropical montane forests. Our objectives include: (a) to investigate the influence of three different time-scale sampling designs – short-term periodic, diel, and hourly – on the perceived VAR patterns; and (b) to provide strategic recommendations for optimal temporal sampling strategies to maximize the utility of limited research resources. By aligning the best practices of sampling strategies with available resources, we believe our findings will promote efficient and effective passive acoustic monitoring, thereby contributing to the conservation of avian communities and terrestrial biodiversity.

## 2. Methods

### 2.1 Study area

This study was conducted in the southern sector of Yushan National Park (YSNP) situated in central Taiwan, encompassing an expanse greater than 100,000 hectares (Underlying data: Figure S1 ( Wu *et al*., 2023)). YSNP is named after Yushan or Jade Mountain, renowned for its highest peak in Northeast Asia with an elevation of 3,952 meters. This national park is pivotal in sustaining high-altitude ecosystems, transitioning from subtropical zones at its base to alpine zones at higher altitudes. The mean annual precipitation recorded is around 3,600 mm. Altitudinal variation influences the average annual temperatures: approximately 20°C at 1,000 meters, around 10°C at 2,500 meters, and roughly 5°C beyond 3,500 meters ( Minister of the Interior, 2022).

### 2.2 Soundscape data collection

For the data collection, 14 PAM stations were deployed within the study area. These stations spanned a distance of approximately 10 kilometers along the Southern Cross-Island Highway, from Meishan (23°15′51″N, 120°49′33″E) to Yakou (23°15′51″N, 120°57′28″E) (Underlying data: Figure S1 ( Wu *et al*., 2023)). The altitude of the initial station, SCIH07, was 1,500 meters, and subsequent stations were set up at 100-meter elevation increments, terminating at an elevation of 2,800 meters with station SCIH20. The distance between any two adjacent stations ranged from 750 and 1,850 meters. This elevation gradient encapsulated four distinctive vegetation types: submontane evergreen broad-leaved forest, montane evergreen broad-leaved forest, montane mixed coniferous-broadleaved forest, and upper montane coniferous forest ( Minister of the Interior, 2022) (Underlying data: Table S1 ( Wu *et al*., 2023)).

Each PAM station was equipped with a Song Meter Mini recorder (Wildlife Acoustic Inc.) designed to capture soundscape data. These devices, anchored to trees at an average height of 1.5 meters, operated continuously throughout the day between March and June 2021. This period corresponds with the breeding season of the region’s montane forest avian species. The recording configurations were set to mono mode, capturing audio in a 16-bit WAV format with a sampling rate of 44.1 kHz. To facilitate subsequent analytical procedures, the recordings were segmented and stored in three-minute durations.

### 2.3 Target species

To gain a comprehensive understanding of individual species’ status within a community, we utilized ecological guilds as our primary criterion for selecting target species. Ding (1993) cataloged 59 avian species recognized as montane forest breeders and categorized them into 12 distinct ecological guilds within YSNP: raptorial carnivores (RC), ground graminivores (GG), ground omnivores (GO), ground insectivores (GI), bush insectivores (BI), tree fruitivores (TF), tree omnivores (TO), tree insectivores (TI), bole gleaners (BG), bole peckers (BP), tree hoverers (TH), and air flycatchers (AF).

To identify our research’s target species, a singular species was selected as a representative from each guild. When a particular guild included more than two species, we used trait data from Tsai *et al*. (2020) regarding Taiwan’s breeding birds to inform our selection. The species that manifested an altitudinal distribution most congruent with our study’s objectives was then chosen.

As a result, we designated 12 bird species as our primary focus, including Collared Owlet ( *Taenioptynx brodiei*), Large-billed Crow ( *Corvus macrorhynchos*), Taiwan Bush Warbler ( *Locustella alishanensis*), Grey-chinned Minivet ( *Pericrocotus solaris*), Taiwan Vivid Niltava ( *Niltava vivida*), Eurasian Nuthatch ( *Sitta europaea*), Taiwan Rosefinch ( *Carpodacus formosanus*), Taiwan Yuhina ( *Yuhina brunneiceps*), Taiwan Shortwing ( *Brachypteryx goodfellowi*), Ashy Wood-Pigeon ( *Columba pulchricollis*), Green-backed Tit ( *Parus monticolus*), and Gray-headed Woodpecker ( *Picus canus*). Each of these species was emblematic of the 12 aforementioned guilds (Underlying data: Table S1 ( Wu *et al*., 2023)).

### 2.4 Vocal detection and performance evaluation

We selected SILIC, an automated wildlife sound identification tool recently developed based on the YOLOv5 object detection model and spectrogram images ( Wu *et al*., 2022), for detecting bird vocalizations in our study. This choice was primarily motivated by two key attributes of SILIC. Firstly, it can recognize the vocalizations of 141 bird species native to Taiwan, encompassing all of our 12 target species. Secondly, SILIC offers a unique capability to detect each vocalization’s exact start and end time within an audio recording at the millisecond level, rather than merely identifying the presence or absence of a certain vocalization within a broad time frame. This feature enables precise computation of the VAR, a crucial metric for our research.

SILIC categorizes sounds into ‘sound classes’ rather than by species. Of the 12 target species we studied, each had 1 to 5 sound classes in SILIC, including ‘song’, ‘call’, ‘drumming’, and ‘unknown’ (a classification with an undetermined function). As song is common during many bird species’ breeding, it was our primary choice. If multiple song classes were provided by SILIC, we consulted experts to select the prevalent one. For species without a song or if the song was understated and hard to discern, we chose a frequently observed sound class. Accordingly, ‘song’ represented 9 of the 12 species, ‘call’ 2, and ‘unknown’ 1. The spectrograms representing the selected sound classes are provided in the Underlying data: Figure S2 ( Wu *et al*., 2023).

We utilized SILIC to extract vocalizations of our target species from the soundscape recordings. Each three-minute recording was segmented into three-second spectrogram clips and analyzed using a one-second sliding window. Due to the overlapping nature of the sliding window, one vocalization might be detected multiple times. For detections of the same species within a single recording, if the intersection area of two overlapping bounding boxes (each bounding box representing a specific vocalization in the spectrogram) divided by the area of the smaller bounding box exceeded 0.25 (Underlying data: Figure S3 ( Wu *et al*., 2023)) or if the intersection area divided by the union area exceeded 0.1 (Underlying data: Figure S4 ( Wu *et al*., 2023)), the two bounding boxes (vocalizations) were combined.

A random sample of 100 detected vocalizations for each species, each tagged with a confidence score (ranging from 0 to 1, indicating the level of certainty that the vocalization belongs to a particular species), was manually reviewed. This set of manually reviewed detections constituted our test dataset for evaluating the performance of SILIC on our soundscape recordings. We created a confusion matrix consisting of four parameters: true positives (TP), true negatives (TN), false positives (FP), and false negatives (FN). Subsequently, we calculated precision as TP/(TP+FP) and recall as TP/(TP+FN). Additionally, we computed the area under the receiver operating characteristic curve (AUC) and the average precision (AP), the latter being equivalent to the area under the Precision-Recall curve. Detailed calculations of these performance metrics can be found in the Underlying data: Appendix S2 ( Wu *et al*., 2023). Finally, we identified the confidence scores corresponding to the maximum F1-score values and designated these as the threshold scores for each species. Detected vocalizations exceeding these confidence score thresholds were classified as positive detections and were incorporated in subsequent analyses.

### 2.5 Temporal sampling designs and statistical analyses

**2.5.1 Environment factors**

As previously stated, bird vocal activity is influenced by multiple factors. In addition to species and seasonality, it is also affected by environmental factors such as climatic conditions, topographic features, and vegetation type. Some of these factors, like topographic features and vegetation type, may remain constant throughout the survey period, while others may vary over time. When investigating the impact of temporal sampling on avian vocalization activity, it is crucial to collect as much information as possible about these environmental factors to comprehensively explore their influence on the VAR. We collected data on each station’s altitude (in meters) and vegetation types (as described in the Soundscape data collection section). Daily climate data, including corrected precipitation (Rainfall; mm), wind speed at two meters (WindSpeed; m/s), temperature at two meters (Temp; °C), relative humidity at two meters (RH; %), and surface pressure (SP; kPa), were downloaded from the NASA/POWER CERES/MERRA2 Native Resolution Daily Data website ( https://power.larc.nasa.gov/data-access-viewer/).

We employed a forward stepwise selection approach to develop four GAMs to evaluate the relationship between the response variable, daily vocal activity rate (VAR_d, representing the quantity of vocalizations per day), and nine exploratory variables. VAR_d was calculated as the total daily vocalization count for each station and bird species separately. The initial model contained only categorical variables ‘Species’ and ‘Vegetation’, as well as their interaction effect. In the second model, a smooth term for ‘Altitude’ for each ‘Species’ was incorporated. The third model included five additional smooth terms for meteorological variables, namely ‘Rainfall’, ‘WindSpeed’, ‘Temp’, ‘RH’, and ‘SP’. Finally, a smooth term for the day of year (DOY) for each ‘Species’ was included in the final model.

As VAR_d is discrete and highly positively skewed count data, we chose a negative binomial distribution function and a log link function. The collinearity of explanatory variables was assessed using variance inflation factors (VIF), with variables demonstrating values greater than five being discarded, following Zuur *et al*. (2007). A Thin Plate Regression Splines (TPRS) smoother was employed for each smooth term as it can capture a wide variety of functional forms, making them suitable for modeling complex nonlinear relationships without having to specify the functional form in advance. The performance of the models was assessed by calculating the deviance explained and Akaike’s Information Criterion (AIC). The analyses were conducted using the “ mgcv” package (version 1.9-0) in R statistical software (version 4.3.1).

**2.5.2 Short-term periodic sampling**

In a single survey season, should there be an insufficient number of ARUs for concurrent recordings across all sampling sites, necessitating rotation, discrepancies in temporal setup could introduce biases. Such inconsistencies might undermine the reliability of subsequent analyses. For example, shifts in breeding statuses over time might influence vocalization frequencies ( Slagsvold, 1977). Thus, during experimental design, it is imperative to target a timeframe wherein temporal variations in avian vocalizations are minimal. Essentially, intervals should be chosen where daily vocal patterns remain sufficiently stable to complete rotation recording within that period. Given the time constraints associated with ARU rotation, reducing deployment durations at individual sites might allow more rotation cycles. Yet, exceedingly short recording spans might risk data bias ( Pérez-Granados *et al*., 2019b). In the segment discussing short-term periodic sampling, we address two facets: (1) Time window: pinpointing intervals throughout the PAM survey season when bird acoustic activity is relatively uniform; and (2) Survey duration: ascertaining the required days for a PAM survey to garner representative data.

For the time window, we employed HCA to group dates with similar vocal activity patterns. We first calculated daily VAR (VAR_d) separately for each station and species, and normalized the data using Z-score normalization to achieve a mean value of zero and a standard deviation of one. Subsequently, we conducted HCA to identify dates with Euclidean distance and Ward’s linkage. The root-mean-square error (RMSE, the square root of the sum of squared distances between any two samples within a cluster divided by the number of samples) and the within-cluster maximum distance (WCMD, the greatest distance between any two samples within a cluster) were computed as indicators of data homogeneity within clusters. The clustering analysis and metric calculations were performed using Python (version 3.10.9), with the SciPy (version 1.10.0) and Scikit-learn (version 1.2.1) packages. Visualization was accomplished using the Matplotlib package (version 3.7.0).

For the survey duration, we utilized the CV of daily VAR (VAR_d) values across various consecutive survey durations as an evaluation metric to assess the influence of different consecutive survey durations on the reliability of VAR_d values. Initially, we defined the consecutive survey durations between one to 14 days for test based on the PAM deployment durations used in previous works ( Machado *et al*., 2017; Wood *et al*., 2020; Metcalf *et al*., 2021; Rumelt *et al*., 2021; Jahn *et al*., 2022). For each duration, starting from day one, we extracted the corresponding data, calculating the VAR_d values and their mean. Upon completion of calculations, we moved forward day by day and repeated the extraction and calculation process for the same duration until all days were covered. Finally, from the obtained average VAR_d values, we calculated their standard deviation and mean. The ratio of these two values yielded the CV. We applied this calculation to each homogeneous cluster obtained from the Time window analysis, allowing a comparative assessment of CV differences across clusters.

**2.5.3 Diel sampling**

To conserve both the storage capacity of audio files and the time required for analysis, recordings are typically sampled during peak vocalization periods of target species within a day. We divided the day into 24-hour segments. Using a GAM, we evaluated the association between an hourly VAR response variable (VAR_h, denoting the quantity of vocalizations per hour) and three explanatory variables: species (categorical), DOY (smooth term), and hour (smooth term). Due to the same rationales applied in the environmental factor analysis, we employed a negative binomial distribution with a log link function and a TPRS smoother. The collinearity of the explanatory variables was evaluated using the VIF; variables with VIF values greater than five were excluded. To represent the 24 hours in a day, a value of 24 basis dimensions (k) was utilized. All analyses were performed using the “mgcv” package (version 1.9-0) in the R statistical software (version 4.3.1).

**2.5.4 Hourly sampling**

As we conducted continuous 24-hour recordings each day, we possessed comprehensive recording data, enabling us to simulate different hourly sampling designs and examine their impacts on the VAR value per minute (VAR_m). We concentrated on two main dimensions: coverage (representing the ratio of time recorded in an hour) and dispersion (indicating the number of recording intervals within an hour, with an X:Y format signifying cycles of X minutes of recording followed by Y minutes of inactivity). Based on seven coverage patterns, we simulated 21 unique sampling combinations for each hour, as elaborated in Table 1. For every species, date, and hour, we derived the mean VAR_m values from both continuous recordings and various sampling strategies, subsequently determining the difference between these values. In the end, we computed the average difference in VAR_m values across different species and sampling designs. A reduced mean difference indicates closer alignment between the VAR_m values from continuous recording and a particular sampling design.

**Table 1.** Hourly sampling combinations from seven coverage designs. This table lists 21 distinct sampling combinations derived from seven temporal coverage designs. ‘Temporal coverage’ indicates the fraction of an hour recorded. In the ‘Sampling combinations’ column, the format X:Y designates cycles of X minutes of recording (ON) succeeded by Y minutes of pause (OFF). These sampling designs were simulated to evaluate their effects on the VAR_m.

Temporal coverage	Sampling combinations
1/2	1:1, 5:5, 15:15, 30:30
1/3	1:2, 5:10, 10:20, 20:40
1/6	1:5, 2:10, 5:25, 10:50
1/10	1:9, 3:27, 6:54
1/15	1:14, 2:28, 4:56
1/30	1:29, 2:58
1/60	1:59

## 3. Results

### 3.1 Vocal detection

Between March 1 and June 30, 2021, spanning 122 days, a total of 789,986 three-minute audio files were collected across 14 sampling stations, approximately totaling 39 thousand hours. During this period, two stations, SCIH11 and SCIH18, failed to record audio files from mid-May to mid-June due to memory card issues. These two stations were subsequently excluded from further analysis. Of the remaining 12 stations, due to deployment scheduling, equipment operation, and battery management issues, at least one station had days where recorded data did not reach 23.5 hours for a total of 10 days. Data from these specific days (i.e., DOY 60–62, 141, 168, and 177–181) were also omitted from further analysis. Hence, data from 12 stations over 112 days continued for subsequent analysis.

We employed SILIC (Version exp29) for automated sound detection. Upon manual inspection by experienced bird surveyors of the 1,200 randomly sampled entries detected, 424 were confirmed as true detections, while 776 were false detections. AUC scores for each species, derived from the test set, ranged from 0.87 to 1.0, and AP scores ranged from 0.85 to 1.0. This demonstrates the excellent detection performance of SILIC within the scope of this study, making it apt for further analysis.

We selected the confidence score at which the precision score for each species was not less than 0.95 as the threshold to minimize the occurrence of false positives. Sound detection results with a confidence score greater than or equal to the threshold were screened for subsequent analysis. In total, 8,202,731 vocalizations from 12 species were detected, with the Taiwan Yuhina having the highest count at 2,863,838, and the Gray-headed Woodpecker the lowest at 23,312. Detailed data on vocalizations detected by SILIC can be found in the Underlying data: a compressed file “VAR_m_all_columns.zip” and a summary as presented in Table S2 ( Wu *et al*. (2023)). For comprehensive information on the test datasets, threshold values, and various performance metrics for each species, please refer to the Underlying data: Table S3 ( Wu *et al*. (2023)). Precision and recall curves are provided in the Underlying data: Figure S5 ( Wu *et al*. (2023)).

### 3.2 Environment factors

Collinearity tests revealed that the variance inflation factor (VIF) for all exploratory variables was below 5, hence all variables were retained for forward stepwise GAM modeling and fitting. When only using species and vegetation types as predictors, the deviance explained is 50.1%. Incorporating altitude as a smoothed term, differentiated by species, increased the deviance explained to 62.9%. Subsequently, by adding five climatic variables as smoothed terms, the deviance explained rose to 68%. Finally, introducing DOY while distinguishing among species further increased the deviance explained to 73.2%. This demonstrates that each variable contributes to the prediction of daily vocal activity rate (VAR_d), as shown in Table 2.

**Table 2.** Model selection results for predicting daily vocal activity rate with GAMs. This table summarizes the outcomes of a forward stepwise variable selection procedure, detailing the Akaike Information Criterion (AIC), adjusted R ^2^, and proportion of deviance explained. The GAM predicts the daily vocal activity rate (vocalizations per day) based on interactions between Species and Vegetation types. The model includes smoothed effects of Altitude (meters, varying by Species), corrected precipitation (Rainfall, mm), wind speed at 2 meters (WindSpeed, m/s), temperature at 2 meters (Temp, °C), relative humidity at 2 meters (RH, %), surface pressure (SP, kPa), and Day of the Year (DOY, varying by species). Weather data were sourced from the NASA/POWER CERES/MERRA2 Native Resolution Daily Data repository, accessible at https://power.larc.nasa.gov/data-access-viewer/. Vegetation data were provided by the Minister of the Interior (2022).

Model (predictors)	AIC	R ^2^(adj.)	Deviance
Null	206430	0	0%
Species * Vegetation	192263	0.358	50.1%
Species * Vegetation + s (Altitude, by=Species)	186863	0.456	62.9%
Species * Vegetation + s (Altitude, by=Species) + s (Rainfall) + s (WindSpeed) + s (Temp) + s (RH) + s (SP)	184246	0.438	68.0%
Species * Vegetation + s (Altitude, by=Species) + s (Rainfall) + s (WindSpeed) + s (Temp) + s (RH) + s (SP) + s (DOY, by=Species)	181310	0.474	73.2%

The model with the lowest AIC, encompassing all nine exploratory variables, was chosen. Most fixed effects and all smoothed terms (including altitude, DOY, and climatic variables) were found to have a significant impact on the predictive capacity of the model, as detailed in the Underlying data: Table S4 ( Wu *et al*., 2023).

While all 12 species exhibited significant correlations between DOY and the VAR_d, the patterns of these relationships were not consistent across species. Some species displayed a strong positive correlation in the early stages of the survey period, which shifted to a pronounced negative correlation in later stages. Conversely, other species demonstrated the opposite pattern (refer to the Underlying data: Figure S6 ( Wu *et al*., 2023)). Similar inconsistencies between species were observed in relation to altitude (see the Underlying data: Figure S7 ( Wu *et al*., 2023)).

When rainfall was less than 40 mm, there was no apparent influence on VAR_d. However, beyond this threshold, VAR_d showed a rapid decrease, with effects diminishing after approximately 60 mm. Wind speeds of up to 3.0 m/s had no discernible effect on VAR_d, but rates declined sharply beyond this speed. For temperatures up to 20°C, VAR_d gradually increased as temperatures rose, with no apparent effects beyond this threshold. Relative humidity had a slight negative effect on VAR_d once it exceeded 80%. As for atmospheric pressure, the impact on VAR_d shifted from negative to positive as pressure moved from low to high. For more detailed information, please refer to the Underlying data: Figure S8 ( Wu *et al*., 2023).

### 3.3 Short-term periodic sampling

**Figure 1.**
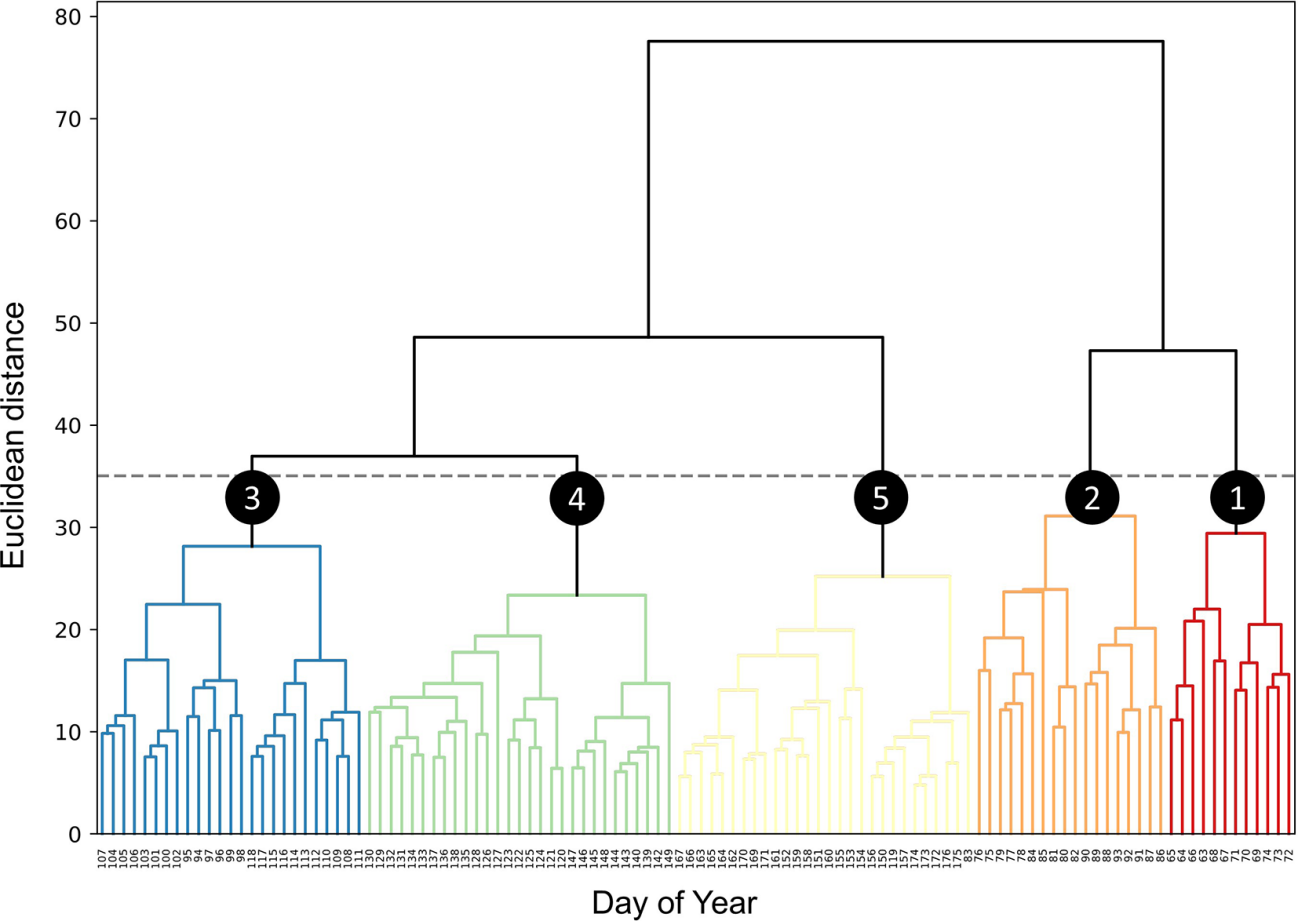
Hierarchical clustering of dates by vocal activity patterns. Dendrogram derived from hierarchical clustering of dates based on daily vocal activity. At a Euclidean distance threshold of 35 (indicated by the grey dashed line), five clusters are discerned (numbered circles).

**Figure 2.**
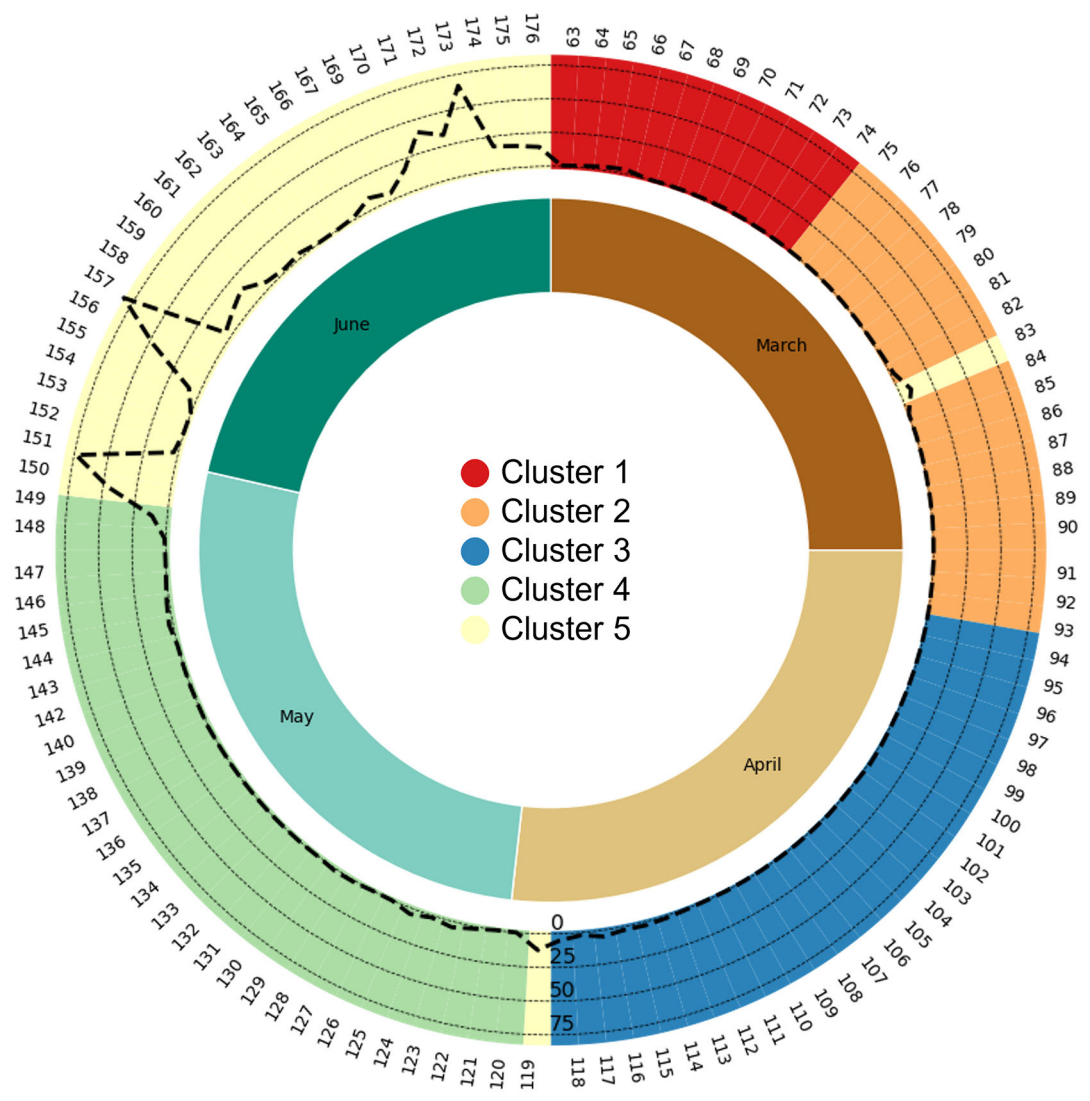
Short-term periodic representation of vocal activity clusters and rainfall. The outer ring indicates vocal activity clusters distinguished by colors: 1 (red), 2 (orange), 3 (blue), 4 (green), and 5 (yellow). Day of Year (DOY) is shown clockwise from the top on the ring's periphery. Within this ring, dashed lines represent rainfall (in mm) from the SCIH13 PAM station, centrally located in the survey area. Two outlier dates in Cluster 5 correspond to rainfall events. The inner ring denotes the months: March to June.

Upon examining the dendrogram derived from HCA ( Figure 1), it becomes evident that at a Euclidean distance of 35, the entire survey period can be partitioned into five distinct clusters. Within each cluster, the VAR_d patterns of different bird species at each PAM station are similar among days within the same cluster but differ from those in other clusters. Notably, Clusters 3, 4, and 5 exhibit substantially lower root-mean-square error (RMSE) and within-cluster maximum distance (WCMD) values compared to Clusters 1 and 2, indicating more homogeneous distributions of VAR_d within these clusters ( Table 3). An observation of the DOY distribution within the five clusters reveals a largely sequential pattern over time, with only two exceptions (DOY 83 and 119) both falling within Cluster 5. Clusters 1 and 2 encompass 12 and 18 days respectively, approximately aligning with the first and latter halves of March. Conversely, Clusters 3, 4, and 5, each containing no fewer than 25 days, roughly correspond to the months of April, May, and June, respectively ( Figure 2).

**Table 3.** Day of year **(**DOY) clusters from hierarchical clustering analysis Using Daily Vocal Activity Rate. This table details the five clusters identified based on daily vocal activity rates. The root-mean-square error (RMSE), within-cluster maximum distance (WCMD), number of days, and specific DOYs are provided for each cluster.

Cluster	RMSE	WCMD	No. of days	DOYs
1	12.48	24.36	12	63~74
2	12.00	24.33	18	75~82, 84~93
3	9.18	18.26	25	94~118
4	8.04	17.55	29	120~149
5	7.78	18.59	28	83, 119, 150~176

**Figure 3.**
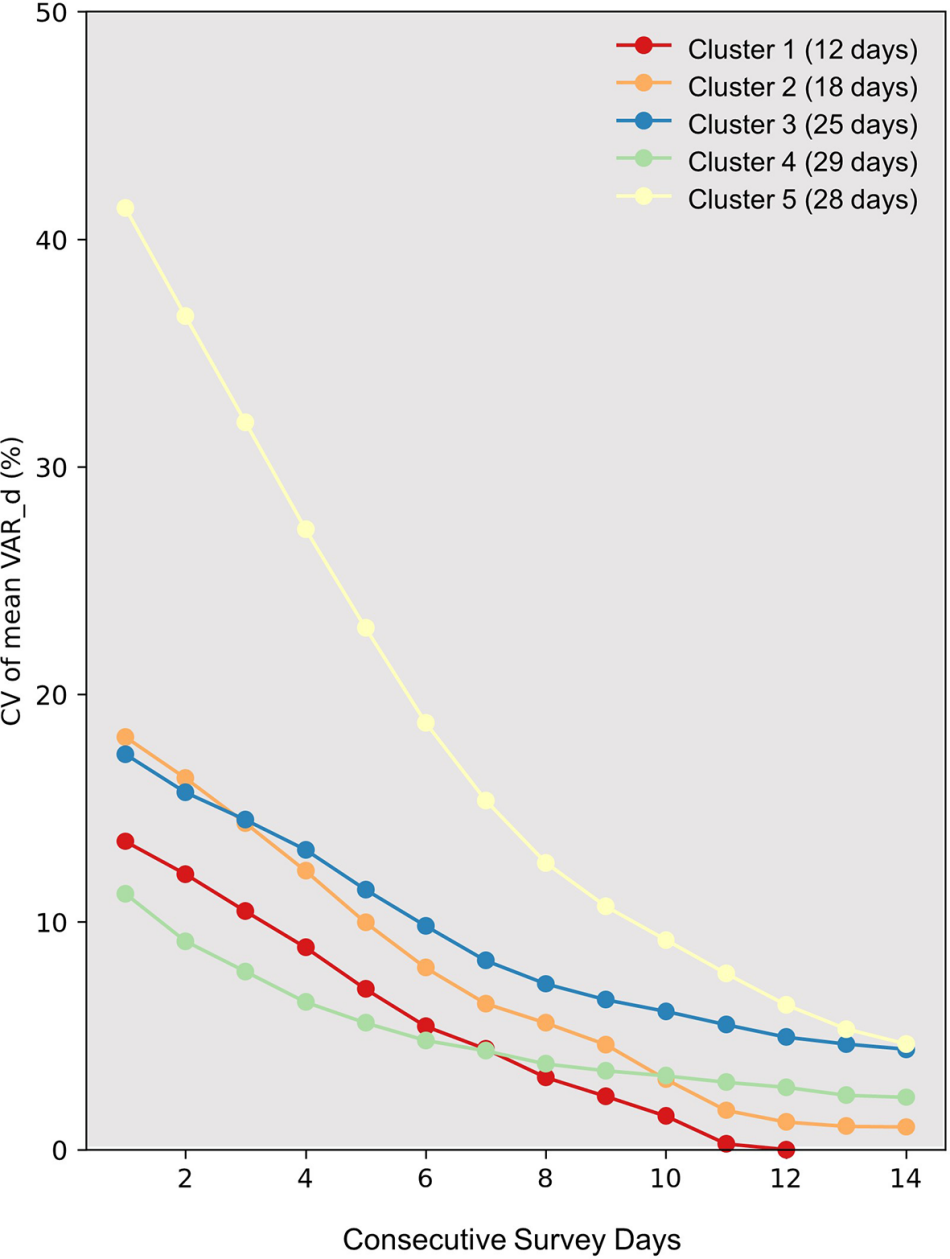
Variation in daily vocal activity rate among clusters. The graph displays the Coefficient of Variation (CV) against consecutive survey days (X-axis) for distinct clusters identified through hierarchical clustering (represented by colored lines). The number of days in each cluster is indicated in parentheses next to the cluster number in the legend. The Y-axis marks the CV values. Interpreting CV values for clusters 1 and 2 should be cautious, particularly for extended survey durations. Their limited days might result in artificially low CV values, potentially underestimating variation.

In Figure 3, the CV of mean VAR_d values spanning one to fourteen consecutive recording days is presented. It is important to note that the sample size (number of days) varies among clusters, particularly with Clusters 1 and 2 having significantly fewer samples compared to the other three clusters. Clusters with a smaller sample size may yield lower CV values compared to those with a larger sample size. Therefore, our discussion focuses primarily on Clusters 3, 4, and 5, which have relatively larger and similar sample sizes. Among the three, Cluster 4 exhibits the lowest CV values, where the decline becomes less pronounced after recording for more than seven days. Conversely, the decrease in CV values for Clusters 3 and 5 persists until recordings reach 14 days, with their final CV values still slightly exceeding that of Cluster 4 when the recording duration is set at seven days.

### 3.4 Diel sampling

Collinearity analysis revealed that the variance inflation factor (VIF) for all explanatory variables was less than 5. Thus, all variables were retained for GAM modeling. The fit of the GAM indicated a deviance explained of up to 80.8% (adjusted R ^2^ = 0.70). All species exhibited a significant influence on VAR_h with respect to the hour (p < 0.001). Dawn and dusk are defined as approximately an hour before and after sunrise and sunset, respectively. Given the study’s duration of four months, sunrise times oscillated between approximately 5 am and 6 am, while sunset times ranged from around 6 pm to 7 pm. Consequently, dawn is represented from 4 am to 7 am, and dusk from 5 pm to 8 pm.

**Figure 4.**
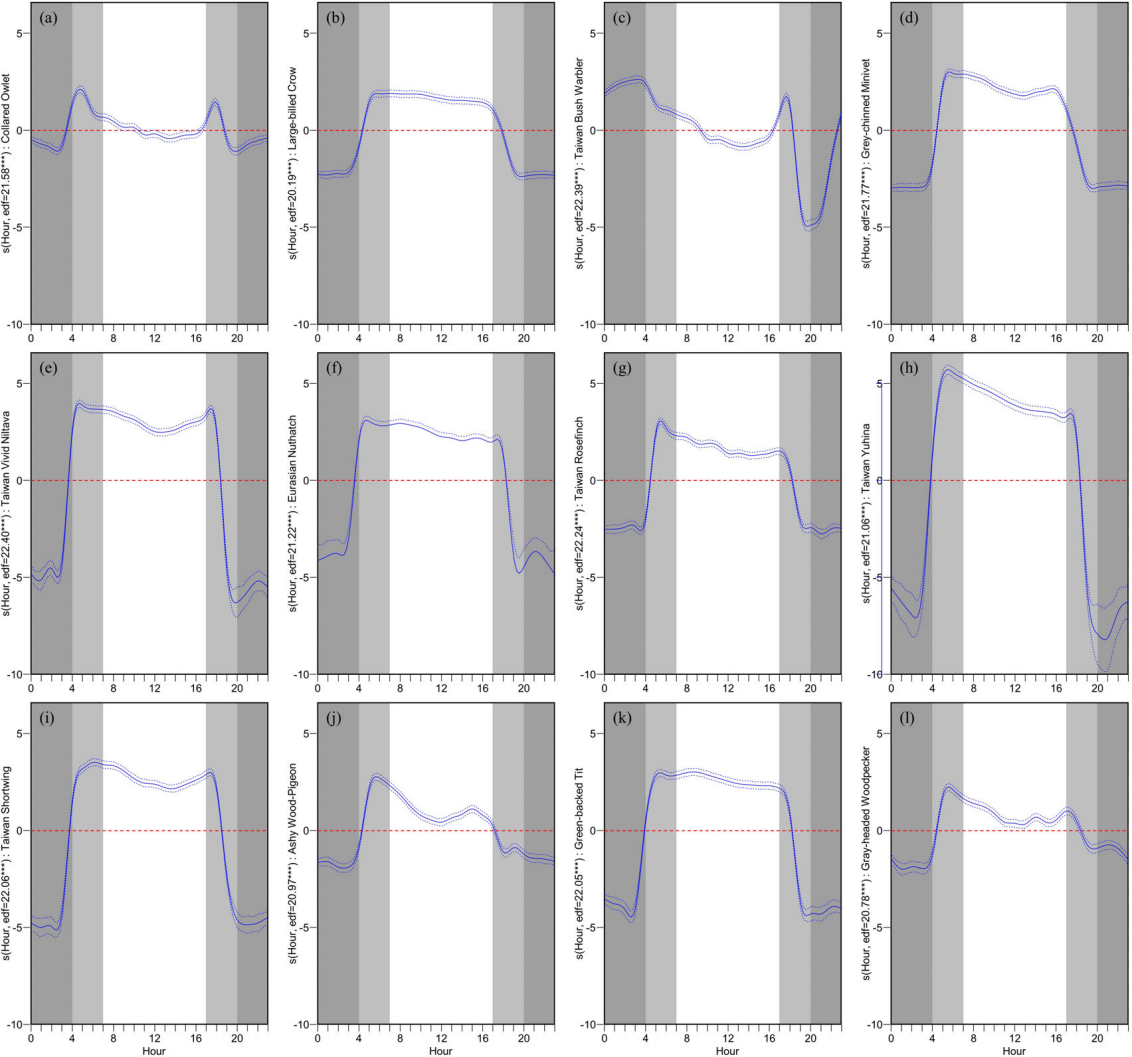
Diurnal patterns of hourly vocal activity for twelve target bird species. Each panel displays the GAM-predicted relationship between the hour of the day and the hourly vocal activity rate (VAR_h) for a specific species: (a) Collared Owlet, (b) Large-billed Crow, (c) Taiwan Bush Warbler, (d) Grey-chinned Minivet, (e) Taiwan Vivid Niltava, (f) Eurasian Nuthatch, (g) Taiwan Rosefinch, (h) Taiwan Yuhina, (i) Taiwan Shortwing, (j) Ashy Wood-Pigeon, (k) Green-backed Tit, and (l) Gray-headed Woodpecker. The y-axis represents the smooth effect of hour on VAR_h for each species. The solid blue line represents the predicted deviation with a 95% confidence interval (blue dashed lines). A reference line is shown at Y=0 (red dashed line). A deep gray shade indicates nighttime, while dawn (approx. 4 AM to 7 AM) and dusk (approx. 5 PM to 8 PM) are highlighted in light gray, reflecting variations due to sunrise and sunset times over the study's four-month span. Accompanying each plot are the estimated degrees of freedom (edf) and significance codes (*** p < 0.001, ** p < 0.01, * p < 0.05).

Observations from Figure 4 regarding the hourly impact on VAR_h reveal that, except for the Collared Owlet and the Taiwan Bush Warbler, the remaining 10 species exhibited a significant positive influence on VAR_h during dawn. This influence gradually declined during the day and swiftly transitioned from a positive to a negative impact at dusk. Throughout the night, a consistent, highly negative influence was observed. The Collared Owlet displayed rapid fluctuations in its influence on VAR_h during dawn and dusk, transitioning from a mild positive effect during the day to a negative one, and maintaining a mildly negative influence at night. The Taiwan Bush Warbler transitioned from a strong negative impact on VAR_h during the late night to a positive one, peaking just before dawn and then declining. During the day, it transitioned from a mild positive to a negative influence, and finally, it exhibited intense fluctuations during dusk, soaring from a negative to a pronounced positive influence before plummeting to a strong negative impact.

### 3.5 Hourly sampling

**Figure 5.**
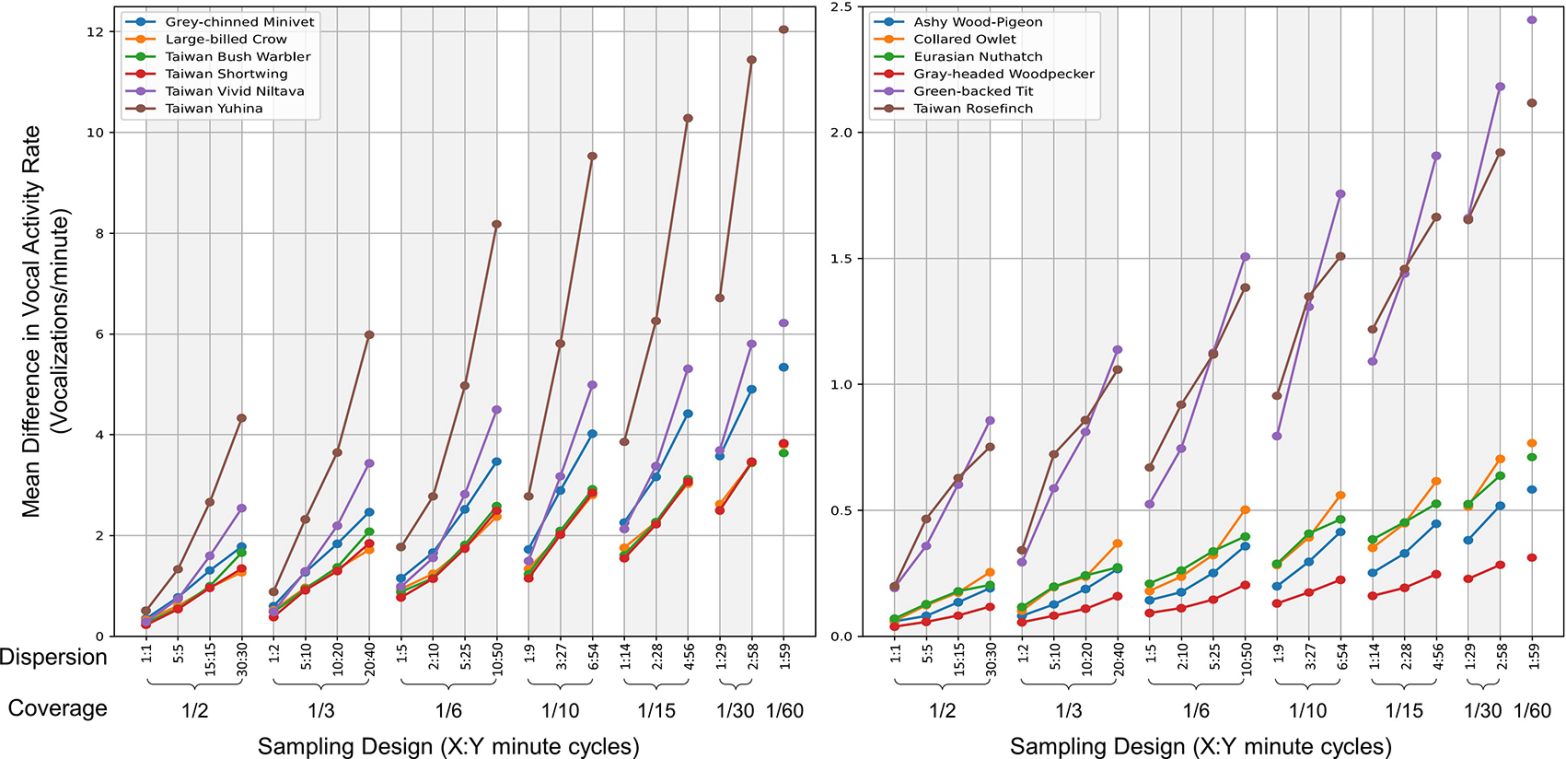
Comparison of vocal activity rates across sampling designs for twelve target bird species. The graph showcases the mean differential VAR_m (vocalizations per minute) between continuous recordings and various sampling methods for each species. Each subplot presents six species differentiated by unique colors. The x-axis lists sampling designs, ordered by decreasing coverage (proportion of the hour recorded) and dispersion (denoted as X:Y, indicating X minutes of recording followed by Y minutes of pause). A reduced mean differential suggests a closer match between the VAR_m from the sampling method and the continuous recording.

Analysis of 21 distinct combinations of coverage and dispersion revealed that higher proportions of recording time, coupled with shorter and more dispersed recording segments, result in VAR_m values from sampling more closely aligning with those from continuous recordings. This trend was consistent across all target species, as illustrated in Figure 5.

## 4. Discussion

In this study, we selected a group of twelve bird species inhabiting subtropical montane forests, each species representing a distinct ecological guild. It is critical to acknowledge that this selection constitutes merely a small fraction of the entire avian community and the expansive soundscape. Moreover, our study is focused exclusively on a single type of vocalization for each species. Given the fact that different vocal types—such as songs versus alarm calls—may reflect different statuses of a species ( Catchpole & Slater, 2003), the selection of vocal type could potentially impact the sampling design we propose. To minimize this effect, where practical, songs were predominantly chosen as the vocal type for the species under study. This approach implies that our findings are particularly tailored to optimize sampling designs for monitoring breeding species populations within the study area. Consequently, researchers should carefully consider the suitability of their monitoring objectives in light of their selected sampling design. Despite these constraints, the extensive data collected in this study provide valuable insights into the considerable variability in avian vocal activity rates across three distinct temporal scales, highlighting the importance of temporal sampling design in studies predominantly utilizing PAM.

### 4.1 Species and environmental factors

Our study elucidates how vocal activity in subtropical forest-dwelling birds is influenced by species, temporal factors, and external environmental conditions. We show that the VAR pattern is strongly affected by individual species’ interaction with vegetation, altitude and DOY. Climate also plays a significant role, impacting VAR across all species. These findings emphasize the significant challenges posed by utilizing PAM to infer the population status and trends of a specific species. These challenges become even more pronounced when the monitoring effort is directed at multi-species bird assemblages, especially when constrained by equipment and time. Consequently, choosing the most appropriate recording sampling design is crucial to ensure data representativeness and comparability.

### 4.2 Short-term periodic sampling

Throughout a bounded time period, like the breeding season examined here, researchers often consider the entire duration as a closed population, executing repeated data collections and comparative analyses within this window ( Baillie, 1991). However, our analysis of short-term periodic sampling revealed pronounced species-specific temporal variations in vocal data over the course of the time sequence. We partitioned the survey season into five clusters, each reflecting relatively consistent vocal patterns. The vocal behaviors within the first two clusters displayed notable intra-cluster variability, potentially attributable to temporal nuances in breeding phases and vocalization rates among different species ( Slagsvold, 1977). Given the observed variability in vocal behaviors early in the season, we recommend extra care when starting acoustic surveys at the beginning of the breeding season. In our study, Clusters 3 and 4 were identified as the optimal periods for acoustic surveys, as these intervals exhibited the lowest variation in vocalization activity across species. Moreover, these consecutive time frames totaled nearly eight weeks, offering greater flexibility in managing the rotation of recording devices.

For intervals characterized by minimal daily vocalization frequency shifts, we propose that a continuous 14-day recording effectively diminishes sampling variance. During phases with even more consistent patterns, such as the fourth cluster identified in this study (circa May), a seven-day recording suffices. Moreover, we advise discarding data from days with rainfall exceeding 40 mm and average wind speeds surpassing 3.0 m/s. These environmental conditions have been empirically demonstrated to considerably dampen vocal activity rates, a phenomenon corroborated in other avian studies ( Vokurková *et al*., 2018; Robbins, 1981).

### 4.3 Diel and hourly sampling

Regarding diel sampling, even though our primary emphasis was on diurnal birds peaking in morning vocalizations, certain species, like the Taiwan Bush Warbler in this study, and approximately 30% of North American birds vocalize nocturnally ( La, 2012). We therefore recommend sampling approximately one hour before and after sunrise to coincide with the morning chorus. If resources permit, recording sessions could commence from midnight to encompass vocal peaks of nocturnal species ( Schaaf *et al*., 2023; Pérez-Granados & Schuchmann, 2020; Odom & Mennill, 2010).

In terms of hourly sampling, our findings align with studies on marine mammals, indicating that longer recording durations combined with shorter, more spaced-out intervals, yield vocal activity rates similar to continuous recordings ( Thomisch *et al*., 2015). The scope of our investigation was confined to examining the effects of dispersion at the minute level, without delving into higher temporal units such as hours or days. This specific focus was dictated by the prevailing limitation that most ARUs are currently only programmable at the minute level. Additionally, there is a noted scarcity in prior research that has formulated dispersion sampling strategies for intervals extending beyond minutes. Investigations in the future, exploring higher temporal scales, might yield further insights. These could potentially enhance the understanding of sampling methodologies that are capable of reducing the duration of recordings, while simultaneously maintaining the integrity and quality of the data collected.

## 5. Conclusions

This study underscores the significance of optimizing temporal and sampling design in PAM from ecological and conservation perspectives. Through such optimization, we not only ensure efficient use of limited resources but also broaden the scope of the monitoring project in terms of temporal, spatial, and taxonomic. Such refinements enhance our understanding of avian community structures and their responses to environmental changes. Based on the findings of this study, within a similar research scope, we recommend the following guidelines for temporal sampling strategies:

1. Conduct acoustic surveys during the mid-breeding season (April to May), where VAR variability is relatively low.
2. Single survey sessions should last a minimum of seven consecutive days, with 14 days being ideal, to substantially reduce sampling variability.
3. Concentrating recording times around the morning (one hour before and after sunrise) can greatly improve detection rates within limited resources. If resources allow, recording can start as early as midnight to include species that peak in vocal activity at night.
4. Employ a schedule of recording for one minute followed by a five-minute rest (a time coverage of 1/6). This schedule, for most bird species, yields data closer to continuous recording compared to a 30-minute recording with a 30-minute rest interval (time coverage of 1/2), while only requiring one-third of the data volume.
5. Avoid recording or using data during weather conditions with rainfall greater than 40 mm, wind speeds exceeding 3 m/s, and temperatures below 20°C, as these significantly reduce VAR.

However, it is essential to emphasize that our study was specifically conducted on 12 breeding bird species within subtropical montane forests. Consequently, applying these findings to other ecosystems or to a broader range of avian taxa demands careful consideration. Moreover, when the focus of monitoring narrows down to a single species, it becomes crucial to devise a sampling strategy that aligns with the distinctive behaviors of that species. We advocate for future studies to build upon our foundational research, venturing into diverse ecological landscapes and including a broader spectrum of bird species.

When temporal sampling enables more economical collection of acoustic data while ensuring its representativeness, researchers around the world will have the opportunity to collaborate in seeking a consistent and cost-effective temporal sampling standard. This not only facilitates cross-dataset research but also supports manageable data sizes for global-scale or decadal long-term data compilations. Such efforts are instrumental in addressing macro-issues like climate change and promoting sustainable development for humanity.

## Data Availability

Zenodo: Underlying data for ‘Evaluating community-wide temporal sampling in passive acoustic monitoring: A comprehensive study of avian vocal patterns in subtropical montane forests’, https://www.doi.org/10.5281/zenodo.8304104 ( Wu *et al*., 2023). This project contains the following underlying data:
• Supplementary Material_v2.pdf. (This file encompasses Figures S1 to S9, Tables S1 to S4, and Appendix S2 which details the calculations for performance metrics.) • VAR_m_all_columns.zip. (This file comprises columns such as site_name, longitude (WGS84), latitude (WGS84), altitude (meters above sea level), vegetation types, date, hour, minute, Day of Year (DOY), scientific_name, and Vocal Activity Rate per minute (VAR_m). The vocalization data is aggregated by site, species, and time (down to the minute). Data are available under the terms of the Creative Commons Attribution 4.0 International license (CC-BY 4.0)
