# Supplementary material for: Evaluating community-wide temporal sampling in passive acoustic monitoring: A comprehensive study of avian vocal patterns in subtropical montane forests
Source: F1000Res. 2024 Jan 23;12:1299. Originally published 2023 Oct 11. [Version 2] doi: 10.12688/f1000research.141951.2 (PMC11036034; doi:10.12688/f1000research.141951.2)
Supplement: Peer review history

# Reviewer report: Reviewer response for version 2

**DOI:** 10.5256/f1000research.161617.r239937

**Published:** 2024-04-22

**Authors:** Jerry S Cole

**Affiliations:** 1 The Institute for Bird Populations, Petaluma, California, USA

Summary: The authors have done their best to address each of the concerns that I raised in my first review of the submission. I thank them for their thoroughness and thoughtful responses. I think the paper has been improved to a level that I feel comfortable approving it for indexing.

Is the work clearly and accurately presented and does it cite the current literature?

Yes

If applicable, is the statistical analysis and its interpretation appropriate?

Yes

Are all the source data underlying the results available to ensure full reproducibility?

Partly

Is the study design appropriate and is the work technically sound?

Yes

Are the conclusions drawn adequately supported by the results?

Yes

Are sufficient details of methods and analysis provided to allow replication by others?

Yes

Reviewer Expertise:

Bioacoustics, ecology, ornithology, automated sound classification

I confirm that I have read this submission and believe that I have an appropriate level of expertise to confirm that it is of an acceptable scientific standard.

# Reviewer report: Reviewer response for version 1

**DOI:** 10.5256/f1000research.155438.r224078

**Published:** 2024-03-04

**Authors:** Bárbara Freitas

**Affiliations:** 1 National Museum of Natural Sciences (Ringgold ID: 16625), Madrid, Community of Madrid, Spain

In the manuscript ‘Evaluating community-wide temporal sampling in passive acoustic monitoring: A comprehensive study of avian vocal patterns in subtropical montane forests’, the authors aimed to investigate the impact of different temporal sampling designs on the detected vocal activity rate of 12 bird species. The authors showed that there is substantial variability in these species’ vocal activity rates across three distinct temporal scales (seasonal, diel, and hourly) and then provided specific recommendations for each one of them and for different environmental conditions.

 Although this study is an interesting case study that provides valuable insights into optimizing temporal sampling designs for Passive Acoustic Monitoring, it needs a more thorough discussion. The recommendations provided appear overly broad and exaggerated, considering the specific scope of this study centred on 12 bird species within subtropical montane forests. The generalizations made may not adequately align with the specific context of this research. This limitation is only briefly mentioned in the conclusion. In this regard, the manuscript could substantially improve by elaborating further on the limitations and potential biases of the study design. Addressing the study's constraints, such as the limited species selection and specific geographical location, would provide a more nuanced understanding of the scope of the conclusions.

 Furthermore, the manuscript would significantly benefit from discussing the potential applicability of the findings in different ecological contexts. Thus, authors should consider expanding upon how these findings might translate to other ecosystems or a more extensive range of avian taxa, or even other classes.

 Finally, some recommendations regarding sampling durations and environmental conditions should be further elucidated. Clarifying the reasoning behind specific recommendations would enhance the applicability and understanding of these suggestions.

**General comments**

 Throughout the manuscript, there is a notable overuse of acronyms, which impedes readability. This hinders comprehension and creates difficulty for readers. Reducing unnecessary acronyms and ensuring consistent explanations for those used is crucial to enhance the overall readability and understanding of the manuscript. By minimizing the reliance on acronyms and providing clear explanations, the text can become more accessible and easier for readers to navigate

 In the abstract, certain acronyms are introduced but not subsequently utilized, contributing to confusion. Please also check if all acronyms are explained within the text.

**Abstract**

 There is some important information missing: the place where the data was collected, in which season, and the number of days of the earliest two segments that showed high variability.

**Introduction**

 The authors state that birds serve as an ‘ideal indicator taxon for monitoring terrestrial biodiversity due to their detectability, identifiability, diversity, widespread distribution, and migratory characteristics’. However, many of these characteristics are also present in other taxa. Emphasis should be given to the detectability and identifiability parts, especially through sound and the availability of automatic detectors for this class.

 The statement regarding the use of sampling lacks references to support it. Authors should explain better and elaborate more on the exogenous and endogenous factors that modulate avian vocal activity. As it is now, it is not clear to the reader.

**Methods**

 The authors do not indicate if this study was or was not preregistered. This must be provided, according to the journal guidelines: "Authors must include a statement to indicate if they did or did not preregister the research with or without a data analysis plan at an independent registry"

 Target species section – it is not clear which criteria the authors followed to define a species as representative of each guild: which parameters were they taking into account? Regarding the selection criteria when two or more species were available for the same guilt, the authors explain that the species ‘that manifested an altitudinal distribution most congruent with’ the study objectives was chosen, but this is vague. The authors should be more specific and explain the criteria with examples. If, for example, high detectability was a factor used to select species, this should be mentioned. Lastly, Table S1 was indicated in the last part of this section but this table does not mention the 12 guilds.

 The reader would benefit from having more information about the SILIC software. For example, the five classes ‘song’, ‘call’, ‘drumming’, and ‘unknown’ are labelled by SILIC or they are just grouped and then labelled by the authors? What is the aim or need of combining bounding boxes of the same species within a single recording?

 It should be indicated the mean of the confidence score of the random sample of 100 detected vocalizations. the definition of AUC should be added as well as an explanation on why this and AP were used.

 The reference for YOLOv5 should be added.

**Results**

 The number of files and equivalent time for the used for subsequent analyses (from 12 stations over 112 days) should be indicated.

**Discussion**

 This section is oversimplified and the recommendations lack robust baseline support.

 The authors made use of words such as behaviors, internal and external factors without really specifying what they refer to.

 The authors suggest sampling around one hour before and after sunrise to capture the morning chorus, even proposing starting recordings at midnight. However, it's crucial to note that these recommendations might lack empirical validation for this particular field site, so the manuscript should highlight the need for further testing or verification.

 Regarding diel sampling, results should be put into context with many more different studies on birds and justification for these findings should be discussed. For example, see (Darras et al. 2019) ^1^

**Minor comments**

 Fig 2- rainfall instead of Corrected Precipitation -  names should be the same throughout the text

 Fig S1 – This figure could be improved by adding a small map of the geographic position of Taiwan in the globe. Please add in the legend what orange means and that the map also depicts the relief.

 Fig S2 – please include axis in each spectrogram. The legend is not necessary to have in all of them (frequency or time), but the axis is fundamental for visualization of the spectrogram.

 Fig S5, S6, S7, S8 – Readability and understanding of the figures could be improved if the name of the species would be written as title of each graph. Also, precision and recall should be explained.

 Table 1 – Please provide a column with the temporal coverage in minutes, to allow for easier understanding.

 Table S3 – Please specify what positive and negative mean. If relative to detections this should be specific in the column name.

Is the work clearly and accurately presented and does it cite the current literature?

Partly

If applicable, is the statistical analysis and its interpretation appropriate?

Yes

Are all the source data underlying the results available to ensure full reproducibility?

No

Is the study design appropriate and is the work technically sound?

Yes

Are the conclusions drawn adequately supported by the results?

Partly

Are sufficient details of methods and analysis provided to allow replication by others?

Partly

Reviewer Expertise:

Bioacoustics, animal communication, passive acoustic monitoring, speciation

I confirm that I have read this submission and believe that I have an appropriate level of expertise to confirm that it is of an acceptable scientific standard, however I have significant reservations, as outlined above.

## References

[1] : Autonomous sound recording outperforms human observation for sampling birds: a systematic map and user guide. *Ecol Appl* .2019;29(6) : 10.1002/eap.1954 e01954 10.1002/eap.1954 31206926

# Reviewer report: Reviewer response for version 1

**DOI:** 10.5256/f1000research.155438.r224421

**Published:** 2024-01-02

**Authors:** Laurel B Symes

**Affiliations:** 1 Cornell University, Ithaca, New York, USA

The goal of this paper is to evaluate how the measured vocal activity rate of birds differs depending on the temporal sampling strategy. Evaluating how detection results differ with sampling strategy is useful, particularly across different species and habitats globally, and can inform decisions about whether it is possible to use a generalized sampling strategies across a range of scenarios, or whether sampling strategies need to vary with season, latitude, habitat type, geography and other factors.

 Overall, the paper seems like a solid evaluation of sampling strategies. Currently, the introduction is strongly focused on the temporal aspects of sampling. In the analysis and figures, the authors devote substantial attention to impacts of environmental co-variates, which may well make sense, but could use some set-up and introduction (or could be downplayed or eliminated). In addition, the authors could provide some additional synthesis/interpretation of their findings in the context of designing PAM-based studies. While the authors provide some specific guidance in the discussion, people seeking actionable information for study design may appreciate even more clear direction (such as headings for sections that discuss recommendations for hourly sampling, diel sampling, etc., and/or a summary in the conclusion that focuses on the key recommendations).

 Major feedback:

*>From the intro “Sampling design can be categorized into four temporal scales: annual, seasonal, diel, and hourly. An annual sampling design implies recording during one or several time periods within a year.”*

 Do you have a reference for the way that these four types of sampling are defined? I would have anticipated that annual sampling compared one year against another (rather than comparing seasons within a year) and that seasonal sampling would have compared one season to another vs “after being operated for a predetermined number of days at each location, the ARU is relocated to a subsequent site” (which to me would generally imply sampling periods shorter than a season).

*>Hourly sampling design can be categorized into coverage (the proportion of recorded time within an hour) and dispersion (the number of recording segments within an hour).*

 Dispersion can affect sampling designs other than hourly as well (for example, if only certain days are sampled). You might consider suggest treating the different temporal scales as one set and then separately discussing dispersion (how thoroughly each of those temporal scales are sampled), whether or not you assess how dispersion of sampling affects outcomes at scales above hourly.

 Some more specifics below:

 Abstract:

  The goal stated in the abstract background (the last sentence) is unclear. Could the authors rephrase the same to indicate clearly what they are attempting to do here? For example, why does the effective temporal sampling design matter?

 You need to introduce what vocal activity rate means prior to discussing the ideal design for VAR data.

 Introduction:

 Having read your introduction – which reads very clearly, I think you might want to rephrase your abstract in the context of trying to identify the right sampling design or approach & why this is important for different ecological research projects/ecosystems/taxonomic groups.

 I think your objectives should be switched – a) trying to identify how sampling designs impact vocal activity rate patterns and b) provide recommendations.

 Based on the key questions asked – your introduction should introduce sampling design followed by a few sentences on vocal activity rate/analysis of acoustic data – and then you could ask your two key questions in your study area. [sampling design -> why this is important for vocal activity rates/patterns -> ask your questions – how does sampling design impact vocal activity rate calculations?]

 Methods:

 Is VAR_d a species-specific measure or is it the total number of vocalizations per day for that site irrespective of the species? Please rephrase/clarify.

 I am unclear on Table 1 – were all these different sampling strategies deployed/implemented in the field or were these post-hoc analysis based on sub-sampling data?

 What does Figure 1 mean ecologically? HCA statistically models Euclidean distance, but what does it mean that day 94 is closely associated with day 110 for example?

 >gender,

 In this context, “sex” may be preferrable to “gender”.

 >Concentrating recordings during specific time, like dawn, often capture more species

 Concentrating recordings during specific time, like dawn, often capture*s* more species

 >For species without a song or the song was

 For species without a song or *if* the song was

 >Secondly, SILIC offers a unique capability to detect each vocalization’s exact start and end time within an audio recording, rather than merely identifying the presence or absence of a certain vocalization within a broad time frame.

 How precisely is this approach able to pinpoint start time? Is this different from other software options that assign call identity within a 3 or 5 second window?

 >In the discussion, you might address how choosing only one vocalization type (generally song) might influence results, vs something like alarm calls, which might have different temporal distribution

 >What is ‘corrected’ precipitation? (vs measured precipitation)

 >Accordingly, ‘song’ represented 9 of the 12 species, ‘call’ 2, and ‘unknown’ 1.

 In supplemental materials, indicate which spectrograms corresponded to which vocalization type

 >spanning 122 working days

 I would remove “working” unless it has specific meaning. (often ‘working’ is used to mean Mon-Fri vs weekend days)

 > Due to the fewer days encompassed by Clusters 1 and 2, when calculating the CV values for a higher number of days, there might be a tendency to obtain relatively lower CV values owing to the smaller sample sizes.

 I found this sentence confusing. I think that it means that clusters 1 and 2 are smaller and should not be compared to clusters that contain higher numbers of days?

 >Figure 2 is visually appealing but may be more complicated to interpret than a horizonal four-month graph. If the figure is retained, the authors might consider having time flow clockwise rather than counterclockwise.

Is the work clearly and accurately presented and does it cite the current literature?

Yes

If applicable, is the statistical analysis and its interpretation appropriate?

Yes

Are all the source data underlying the results available to ensure full reproducibility?

Yes

Is the study design appropriate and is the work technically sound?

Yes

Are the conclusions drawn adequately supported by the results?

Yes

Are sufficient details of methods and analysis provided to allow replication by others?

Yes

Reviewer Expertise:

Bioacoustics, biodiversity, speciation

I confirm that I have read this submission and believe that I have an appropriate level of expertise to confirm that it is of an acceptable scientific standard, however I have significant reservations, as outlined above.

# Response

**Published:** 2024-01-25

**Authors:** Shih-Hung Wu

**Affiliations:** Biological Sciences, National Sun Yat-sen University, Kaohsiung, Kaohsiung City, Taiwan

Dear Dr. Laurel B Symes,

 Thank you for your insightful review of our manuscript. We value your comments on evaluating vocal activity rates and sampling strategies across various species and habitats. In response to your suggestions, we have revised the manuscript accordingly and provide detailed responses to each of your comments below:

 The goal of this paper is to evaluate how the measured vocal activity rate of birds differs depending on the temporal sampling strategy. Evaluating how detection results differ with sampling strategy is useful, particularly across different species and habitats globally, and can inform decisions about whether it is possible to use a generalized sampling strategies across a range of scenarios, or whether sampling strategies need to vary with season, latitude, habitat type, geography and other factors.

 Overall, the paper seems like a solid evaluation of sampling strategies. Currently, the introduction is strongly focused on the temporal aspects of sampling. In the analysis and figures, the authors devote substantial attention to impacts of environmental co-variates, which may well make sense, but could use some set-up and introduction (or could be downplayed or eliminated). In addition, the authors could provide some additional synthesis/interpretation of their findings in the context of designing PAM-based studies. While the authors provide some specific guidance in the discussion, people seeking actionable information for study design may appreciate even more clear direction (such as headings for sections that discuss recommendations for hourly sampling, diel sampling, etc., and/or a summary in the conclusion that focuses on the key recommendations).

- We have included a checklist of considerations in the Conclusions section to offer specific guidance for researchers conducting PAM studies in similar domains. (Section 5, Para. 1, Line 4)

Major feedback:

*>From the intro “Sampling design can be categorized into four temporal scales: annual, seasonal, diel, and hourly. An annual sampling design implies recording during one or several time periods within a year.”*

 Do you have a reference for the way that these four types of sampling are defined? I would have anticipated that annual sampling compared one year against another (rather than comparing seasons within a year) and that seasonal sampling would have compared one season to another vs “after being operated for a predetermined number of days at each location, the ARU is relocated to a subsequent site” (which to me would generally imply sampling periods shorter than a season).

- Indeed, we recognize that our use and definitions of 'annual sampling' and 'seasonal sampling' in the study may have been confusing for readers. We have decided to revise these terms to 'intra-annual sampling' and 'short-term periodic sampling' to avoid such ambiguity. (Abstract - Methods; Section 1, Para. 3, Line 2; Section 1, Para. 4, Line 1; Section 2.5.2, Para. 1, Line 8; Section 3.3; Section 4.2, Para. 1, Line 3)

*>Hourly sampling design can be categorized into coverage (the proportion of recorded time within an hour) and dispersion (the number of recording segments within an hour).*

 Dispersion can affect sampling designs other than hourly as well (for example, if only certain days are sampled). You might consider suggest treating the different temporal scales as one set and then separately discussing dispersion (how thoroughly each of those temporal scales are sampled), whether or not you assess how dispersion of sampling affects outcomes at scales above hourly.

- The scope of our investigation was confined to examining the effects of dispersion at the minute level, without delving into higher temporal units such as hours or days. This specific focus was dictated by the prevailing limitation that most ARUs are currently only programmable at the minute level. Additionally, there is a noted scarcity in prior research that has formulated dispersion sampling strategies for intervals extending beyond minutes. Investigations in the future, exploring higher temporal scales, might yield further insights. These could potentially enhance the understanding of sampling methodologies that are capable of reducing the duration of recordings, while simultaneously maintaining the integrity and quality of the data collected. We have added a section in the Discussion regarding the exploration of dispersion sampling strategies across different temporal scales. (Section 4.3, Para. 2, Line 3)

Some more specifics below:

 Abstract:

 The goal stated in the abstract background (the last sentence) is unclear. Could the authors rephrase the same to indicate clearly what they are attempting to do here? For example, why does the effective temporal sampling design matter?

 You need to introduce what vocal activity rate means prior to discussing the ideal design for VAR data.

- We have supplemented the abstract with a definition of VAR and its significance in the context of our study. (Abstract - Background)

Introduction:

 Having read your introduction – which reads very clearly, I think you might want to rephrase your abstract in the context of trying to identify the right sampling design or approach & why this is important for different ecological research projects/ecosystems/taxonomic groups.

- We have rewritten the background section of the abstract to clearly articulate the primary objectives of our study. (Abstract - Background)

I think your objectives should be switched – a) trying to identify how sampling designs impact vocal activity rate patterns and b) provide recommendations.

- We have made revisions in accordance with your suggestions. (Section 1, Para. 8, Line 2)

Based on the key questions asked – your introduction should introduce sampling design followed by a few sentences on vocal activity rate/analysis of acoustic data – and then you could ask your two key questions in your study area. [sampling design -> why this is important for vocal activity rates/patterns -> ask your questions – how does sampling design impact vocal activity rate calculations?]

- We have made revisions in accordance with your suggestions. (Section 1, Para. 4, Line 4; Para. 5, Line 4; Para. 6, Line 5)

Methods:

 Is VAR_d a species-specific measure or is it the total number of vocalizations per day for that site irrespective of the species? Please rephrase/clarify.

- VAR_d was calculated as the total daily vocalization count for each station and bird species separately. We have added a section to make the narrative more comprehensive and complete. (Section 2.5.1, Para. 2, Line 3)

I am unclear on Table 1 – were all these different sampling strategies deployed/implemented in the field or were these post-hoc analysis based on sub-sampling data?

- Our study is based on continuous field recording data, and it simulates the outcomes and impacts of data acquisition under different temporal sampling scenarios. We have added explanatory text in the relevant sections to clarify this approach. (Section 2.5.4, Para. 1, Line 1; Explanation of Table 1.)

What does Figure 1 mean ecologically? HCA statistically models Euclidean distance, but what does it mean that day 94 is closely associated with day 110 for example?

- Within each cluster, the VAR_d patterns of different bird species at each PAM station are similar among days within the same cluster but differ from those in other clusters.  We have added explanatory text in the relevant section. (Section 3.3, Para. 1, Line 2)

>gender,

 In this context, “sex” may be preferrable to “gender”.

- Revised. (Section 1, Para. 7, Line 5)

>Concentrating recordings during specific time, like dawn, often capture more species

 Concentrating recordings during specific time, like dawn, often capture*s* more species

- Revised. (Section 1, Para. 7, Line 13)

>For species without a song or the song was

 For species without a song or *if* the song was

- Revised. (Section 2.4, Para. 2, Line 4)

>Secondly, SILIC offers a unique capability to detect each vocalization’s exact start and end time within an audio recording, rather than merely identifying the presence or absence of a certain vocalization within a broad time frame.

 How precisely is this approach able to pinpoint start time? Is this different from other software options that assign call identity within a 3 or 5 second window?

- SILIC can detect each vocalization at the millisecond level. We have made revisions in Section 2.4, Para. 1, Line 4.

>In the discussion, you might address how choosing only one vocalization type (generally song) might influence results, vs something like alarm calls, which might have different temporal distribution

- We have revised the discussion segment focusing on the potential impacts of species and vocalization type on our research findings. (Section 4, Para. 1, Line 1)

>What is ‘corrected’ precipitation? (vs measured precipitation)

- The corrected precipitation from NASA/POWER CERES/MERRA2 Native Resolution Daily Data is estimated through algorithms (climate models) that interpret satellite observations. These observations are then often adjusted or 'corrected' using various atmospheric data and models to provide more accurate estimates.

>Accordingly, ‘song’ represented 9 of the 12 species, ‘call’ 2, and ‘unknown’ 1.

 In supplemental materials, indicate which spectrograms corresponded to which vocalization type

- We have added information on the type of vocalization to each corresponding spectrogram. (Supplementary Material_v2.pdf)

>spanning 122 working days

 I would remove “working” unless it has specific meaning. (often ‘working’ is used to mean Mon-Fri vs weekend days)

- Revised. (Section 3.1, Para. 1, Line 1)

> Due to the fewer days encompassed by Clusters 1 and 2, when calculating the CV values for a higher number of days, there might be a tendency to obtain relatively lower CV values owing to the smaller sample sizes.

 I found this sentence confusing. I think that it means that clusters 1 and 2 are smaller and should not be compared to clusters that contain higher numbers of days?

- We have revised our discussion to more accurately address the differences in the CV values among clusters with varying sample sizes. (Section 3.3, Para. 2, Line 1)

>Figure 2 is visually appealing but may be more complicated to interpret than a horizonal four-month graph. If the figure is retained, the authors might consider having time flow clockwise rather than counterclockwise.

- We have adjusted the figure to rotate clockwise, and we have also included Figure S9 in the Supplementary Material, which presents the data with a horizontal time axis. (Figure 2; Figure S9)

# Reviewer report: Reviewer response for version 1

**DOI:** 10.5256/f1000research.155438.r224082

**Published:** 2023-12-19

**Authors:** Jerry S Cole

**Affiliations:** 1 The Institute for Bird Populations, Petaluma, California, USA

Summary: The authors have produced a timely and well written article that helps to further advance our understanding of how VAR differs across species and in response to environmental conditions. I particularly enjoyed the section that used clustering to divide the full sampling season into distinct segments. It would be nice to see a bit more development of the discussion section, for instance suggestions for what additional avenues researchers might explore other than just replicating this study with other species in other locations. Thanks for the interesting read!

 Data Availability:

 Not sure if I missed this but I don't see a location where the raw data - for instance the output summary of number of bird detections per minute for each species on a given sampling day and hour. I assume that at a minimum the raw output from the classifier would be needed to reproduce the data analyses reported here. I see the supplementary material with plots and overall number of detections, but that would not be enough to reproduce the statistical analyses.

 _________

 Introduction

 I enjoyed this introduction. Does a nice job to lead in to the aims of the study.

 Paragraph (P) 3 - Might consider saying "more frequent vocalization" rather than "enhanced vocalization" to be more specific about what exactly is meant - if by "enhanced" you mean "more frequent and louder".

 ____________

 Results

 Section 3.1, P2 - Does "1,200 randomly sampled entries" mean detections by SILIC, or just random sections of the recordings? I think this is probably clarified later in the sentence, but something to think about.

 Fig. 1 and 2 - Really enjoyed this method of defining distinct sampling periods through a cluster analysis.

 __________

 Discussion

 General - Should researchers analyze data from only a single survey season cluster period? If so, which period?And what does this mean for multi-species analysis?

 P2 - Not sure what "interplay of internal ..." means. Does that mean the biology of the bird is partly responsible for differing VAR?

 __________

 Conclusions

 Seems like a good way to wrap it up. I do wonder about how this might apply to a full community of birds, but I suppose that is beyond the scope of this work.

Is the work clearly and accurately presented and does it cite the current literature?

Yes

If applicable, is the statistical analysis and its interpretation appropriate?

Yes

Are all the source data underlying the results available to ensure full reproducibility?

Partly

Is the study design appropriate and is the work technically sound?

Yes

Are the conclusions drawn adequately supported by the results?

Yes

Are sufficient details of methods and analysis provided to allow replication by others?

Yes

Reviewer Expertise:

Bioacoustics, ecology, ornithology, automated sound classification

I confirm that I have read this submission and believe that I have an appropriate level of expertise to confirm that it is of an acceptable scientific standard, however I have significant reservations, as outlined above.

# Response

**Published:** 2024-01-24

**Authors:** Shih-Hung Wu

**Affiliations:** Biological Sciences, National Sun Yat-sen University, Kaohsiung, Kaohsiung City, Taiwan

Dear Dr. Jerry S. Cole,

 We greatly appreciate your positive remarks and thoughtful suggestions, which will be instrumental in refining our paper. Here are our responses aligned with your comments:

 Summary: The authors have produced a timely and well written article that helps to further advance our understanding of how VAR differs across species and in response to environmental conditions. I particularly enjoyed the section that used clustering to divide the full sampling season into distinct segments. It would be nice to see a bit more development of the discussion section, for instance suggestions for what additional avenues researchers might explore other than just replicating this study with other species in other locations. Thanks for the interesting read!

- We have added a visionary suggestion at the end of the 'Conclusions' section, where we express our hope for researchers worldwide to collaboratively develop a universal standard for temporal sampling. This initiative aims to enhance the transferability of PAM data and to bolster the capability for data integration and analysis on a global scale. (Section 5, Para. 3)

Data Availability:

 Not sure if I missed this but I don't see a location where the raw data - for instance the output summary of number of bird detections per minute for each species on a given sampling day and hour. I assume that at a minimum the raw output from the classifier would be needed to reproduce the data analyses reported here. I see the supplementary material with plots and overall number of detections, but that would not be enough to reproduce the statistical analyses.

- Thank you for the reminder. We have now updated the compressed file 'VAR_m_all_columns.zip' in the "Underlying Data" to include the data on the number of calls per minute for each species at each PAM station, along with the associated environmental variable data. (Section 3.1, Para. 3, Line 5)

_________

 Introduction

 I enjoyed this introduction. Does a nice job to lead in to the aims of the study.

 Paragraph (P) 3 - Might consider saying "more frequent vocalization" rather than "enhanced vocalization" to be more specific about what exactly is meant - if by "enhanced" you mean "more frequent and louder".

- We have revised our manuscript in accordance with your suggestions. (Section 1, Para. 3, Line 3)

____________

 Results

 Section 3.1, P2 - Does "1,200 randomly sampled entries" mean detections by SILIC, or just random sections of the recordings? I think this is probably clarified later in the sentence, but something to think about.

- As detailed in Section 2.4, Para. 4, for the evaluation of the SILIC model, we randomly selected 100 detections per species from the results identified by SILIC. Each sample includes a confidence score assigned by SILIC. This approach allows us to assess the performance of the SILIC model and to determine an appropriate confidence score threshold for each bird species relevant to our study. Subsequently, we filtered the total detection results to include only those with a confidence score equal to or exceeding the set threshold score. This process provided us with the necessary data on bird vocal activity for our research. We have revised the text to make the meaning more complete and clear. (Section 3.1, Para. 2, Line 2)

Fig. 1 and 2 - Really enjoyed this method of defining distinct sampling periods through a cluster analysis.

- Thank you for acknowledging the validity of our analytical approach.

__________

 Discussion

 General - Should researchers analyze data from only a single survey season cluster period? If so, which period?And what does this mean for multi-species analysis?

- In our study, Clusters 3 and 4 were identified as the optimal periods for acoustic surveys, as these intervals exhibited the lowest variation in vocalization activity across species. Moreover, these consecutive time frames totaled nearly eight weeks, offering greater flexibility in managing the rotation of recording devices. We have added an explanatory text in Section 4.2, Para. 1, Line 8.

P2 - Not sure what "interplay of internal ..." means. Does that mean the biology of the bird is partly responsible for differing VAR?

- We wish to clarify that vocal activity is influenced by a multitude of factors including species, temporal aspects, and external environmental conditions. We have accordingly revised the wording of this paragraph in Section 4.1, Para. 1, Line 1.

__________

 Conclusions

 Seems like a good way to wrap it up. I do wonder about how this might apply to a full community of birds, but I suppose that is beyond the scope of this work.

- We intend to continue exploring the impact of temporal sampling on other species and environments in future work. Additionally, we look forward to results shared by researchers from other regions, which would contribute to the development of more broadly applicable universal standards.
